# The emerging role of autophagy and mitophagy in tauopathies: From pathogenesis to translational implications in Alzheimer’s disease

**DOI:** 10.3389/fnagi.2022.1022821

**Published:** 2022-10-17

**Authors:** Xiaolan Liu, Meng Ye, Liang Ma

**Affiliations:** ^1^Wuhan Mental Health Center, Wuhan, China; ^2^Wuhan Hospital for Psychotherapy, Wuhan, China

**Keywords:** Alzheimer’s disease, tau protein, autophagy, mitophagy, neuroinflammation, prevention and treatment

## Abstract

Alzheimer’s disease (AD) is the most prevalent neurodegenerative disease, affecting more than 55 million individuals worldwide in 2021. In addition to the “amyloid hypothesis,” an increasing number of studies have demonstrated that phosphorylated tau plays an important role in AD pathogenesis. Both soluble tau oligomers and insoluble tau aggregates in the brain can induce structural and functional neuronal damage through multiple pathways, eventually leading to memory deficits and neurodegeneration. Autophagy is an important cellular response to various stress stimuli and can generally be categorized into non-selective and selective autophagy. Recent studies have indicated that both types of autophagy are involved in AD pathology. Among the several subtypes of selective autophagy, mitophagy, which mediates the selective removal of mitochondria, has attracted increasing attention because dysfunctional mitochondria have been suggested to contribute to tauopathies. In this review, we summarize the latest findings on the bidirectional association between abnormal tau proteins and defective autophagy, as well as mitophagy, which might constitute a vicious cycle in the induction of neurodegeneration. Neuroinflammation, another important feature in the pathogenesis and progression of AD, has been shown to crosstalk with autophagy and mitophagy. Additionally, we comprehensively discuss the relationship between neuroinflammation, autophagy, and mitophagy. By elucidating the underlying molecular mechanisms governing these pathologies, we highlight novel therapeutic strategies targeting autophagy, mitophagy and neuroinflammation, such as those using rapamycin, urolithin, spermidine, curcumin, nicotinamide, and actinonin, for the prevention and treatment of AD.

## Introduction

Alzheimer’s disease (AD) is the most prevalent age-associated neurodegenerative disease and is characterized by progressive memory loss combined with cognitive impairment (DeTure and Dickson, 2019). It is estimated that approximately 57.4 million individuals were living with AD globally in 2019 (GBD 2019 Dementia Forecasting Collaborators, 2022). The main pathological features of AD include brain atrophy, neuronal loss, intracellular neurofibrillary tangles (NFTs) composed of hyperphosphorylated tau protein, and accumulation of amyloid-beta (Aβ) in cerebral vessels and brain parenchyma (DeTure and Dickson, 2019; Tiwari et al., 2019; Ma et al., 2021). Discussions of the underlying molecular mechanisms of AD are ongoing. According to the amyloid hypothesis proposed in the 1990s, Aβ may play a major role in the pathogenesis of AD (Hardy and Higgins, 1992; Selkoe and Hardy, 2016). However, several Aβ antibodies and BACE1 inhibitors developed by various pharmaceutical companies aiming to decrease Aβ levels have failed in the third phase of clinical trials (van Dyck, 2018; Avgerinos et al., 2021; Jeremic et al., 2021). Thus, tau, another pathological protein in the brains of patients with AD, has attracted increasing attention. Tau is a microtubule-associated protein (MAP) abundant in neurons of the central and peripheral nervous systems of vertebrates (Wang and Mandelkow, 2016). Diseases characterized by insoluble deposits of aggregated tau proteins in neurons and glial cells are known as tauopathies (Rojas and Boxer, 2016). Tau-dependent cytotoxic mechanisms are prevalent in neurodegenerative diseases and involve tau hyperphosphorylation, aggregation and other factors affecting tau physiology and pathology, such as autophagy, mitophagy, and neuroinflammation (Leng and Edison, 2021).

Deficient autophagy and mitochondrial autophagy (mitophagy) in neurodegenerative diseases, particularly AD, have attracted increasing attention as pathogenesis mechanisms as well as potential therapeutic targets (Kerr et al., 2017; Mizushima and Levine, 2020). Unlike other cell types, the normal function of neurons, which exhibits little or no division, relies heavily on autophagy, through which the damaged organelles and misfolded proteins can be successfully removed (Menzies et al., 2017). Similarly, mitophagy is essential for the degradation of dysfunctional mitochondria and the maintenance of mitochondrial homeostasis. Owing to the high energy demands of neurons, mitophagy is considered essential for neuronal energy metabolism (Lou et al., 2020). Additionally, persistent activation of astrocytes and microglia has been observed in the brains of patients with AD in the early stages of the disease, suggesting that neuroinflammation plays a pivotal role in the onset and development of AD (Leng and Edison, 2021). Although defective autophagy, mitophagy, and elevated neuroinflammation have been observed in AD, crosstalk between abnormal tau and autophagy, as well as mitophagy, and the relationship between neuroinflammation and these processes has not been fully elucidated (Green et al., 2011). Understanding these aspects will help further explore disease pathogenesis and investigate potential targets for AD treatment.

In this review, we hypothesized that autophagy and mitophagy could act both upstream and downstream of tauopathies and neuroinflammation, and all these bidirectional interactions might constitute a vicious cycle that enhance neurodegeneration in AD. We first discussed the function of tau, autophagy, and mitophagy in healthy neurons and their dysfunction in tauopathies and then summarized the crosstalk between abnormal tau protein and defective autophagy as well as mitophagy. Relationships between neuroinflammation and autophagy, mitophagy, and tau proteins were also discussed. These bidirectional interactions may constitute a vicious cycle that induces neurodegeneration. Finally, we highlighted current therapeutic strategies targeting autophagy, mitophagy, and neuroinflammation for the prevention and treatment of AD.

## Tau and autophagy/mitophagy

### Physiological and pathological role of tau protein

Tau can polymerize tubulin into microtubules, a process involved in the maintenance of intricate neuronal cellular microstructures, such as microtubule assembly and stability (Guo et al., 2017). In physiological settings, more than 90% of tau proteins are coupled to microtubules in irregularly coiled formations, where they perform crucial roles in axonal transport, cell polarity, and neurotransmission (Zeng et al., 2021). Axonal transport is essential for the efficient movement of neuronal organelles, lipids, proteins, nucleic acids, and synaptic vesicles (Morris et al., 2020). Through microtubule binding, tau proteins are involved in the regulation of axonal transport (Wang et al., 2015). Tau isoforms, mutations, post-translational modifications, and N- or C-terminal truncations greatly affect the function of the tau protein, among which tau phosphorylation is the most prominent regulator (Avila et al., 2004). Under pathological conditions, tau proteins may undergo hyperphosphorylation (Liu et al., 2006). Tau phosphorylation is regulated by several kinases, including glycogen synthase kinase 3 (GSK3β) and cyclin-dependent kinase 5 (CDK5) (Drummond et al., 2020). Hyperphosphorylation of tau may result from an imbalance in the activity of specific tau kinases and phosphatases, which impairs the binding affinity of tau to microtubules and leads to loss of function in microtubule stabilization and axonal transport (Simic et al., 2016). The separation of hyperphosphorylated tau protein from microtubules leads to an abnormal increase in its intracellular concentration. The accumulated cytoplasmic concentration of tau increases its susceptibility to misfolding and promotes the formation of aggregates rich in β-sheet structures, ultimately leading to the formation of NFTs in neurons (Goedert et al., 2017; Vaquer-Alicea and Diamond, 2019).

Soluble tau oligomers, which occur before NFT formation, might be a major cause of neurodegeneration (Forloni et al., 2016). Evidence suggests that tau oligomers disrupt mitochondria by decreasing the synaptic vesicle-associated proteins synaptophysin and septin-11, and by decreasing the levels of NADH-ubiquinone oxidoreductase and electron transport chain complex I (Wang et al., 2020; John and Reddy, 2021).

The ubiquitin-proteasome and the autophagy-lysosome pathways are the two major pathways for intracellular protein degradation (Varshavsky, 2017). Under physiological conditions, misfolded tau proteins and aggregates can be completely degraded by the ubiquitin-proteasome and the autophagy-lysosome pathways (Wang and Mandelkow, 2016). However, under pathological conditions, the substantial increase in hyperphosphorylated tau proteins exerts considerable pressure on the intracellular scavenging system, leading to disturbed protein homeostasis and the deposition of NFTs (Wegmann et al., 2021).

### Maintenance of neuronal homeostasis by autophagy/mitophagy and their dysfunction in Alzheimer’s disease

Autophagy is a process that maintains healthy cells, organelles, proteins, and nutrient homeostasis in living organisms (Mizushima and Levine, 2020). Three types of autophagy are observed in mammalian cells depending on the mode of substrate delivery: macroautophagy, chaperone-mediated autophagy, and microautophagy (Mizushima and Komatsu, 2011). The transportation mode of the target cargo to the lysosome for degradation distinguishes several autophagy types (Kim and Lee, 2014). In macroautophagy, a double-membrane vesicle known as an autophagosome engulfs its targets by isolating a portion of the cytoplasm (Feng et al., 2014). The autophagosomal membrane fuses with lysosomes to form autophagic vesicles, the contents of which are degraded by lysosomal proteases (Griffey and Yamamoto, 2022). In microautophagy, lysosomes directly phagocytose and degrade cytoplasmic components *via* membrane invagination (Fleming et al., 2022). In contrast, in chaperone-mediated autophagy (CMA), chaperone proteins translocate target proteins to the lysosomal receptor and subsequently pass the lysosomal membrane to enter the lysosome (Kaushik and Cuervo, 2018).

Low levels of autophagy occur in cells to maintain homeostasis. However, various life-threatening events, such as hypoxia, nutrient depletion, exposure to reactive oxygen species (ROS), microbial invasion, organelle damage, and excessive accumulation of aggregated proteins, such as tau, can enhance autophagic activity (Maiuri et al., 2007; Choi et al., 2013). Although neuronal autophagy is less common, the normal development and function of the central nervous system (CNS) are more dependent on autophagy compared to other tissues (Sumitomo and Tomoda, 2021). In neurons, autophagy is essential for the maintenance of cellular homeostasis and participates in multiple neuron-specific functions, such as axon guidance, synaptic transmission, proper neuronal connectivity, and neural stem cell development (Menzies et al., 2017; Stavoe and Holzbaur, 2019). Autophagic dysfunction is considered a critical factor contributing to neurodegeneration (Fleming et al., 2022).

**FIGURE 1:**
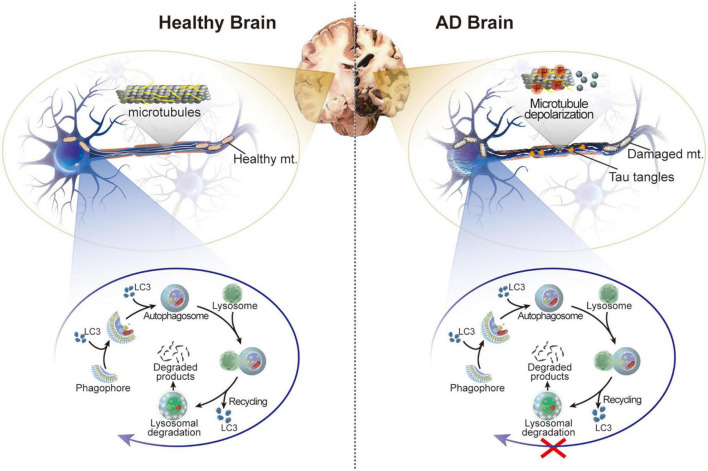
Schematic illustration of the healthy brain and brain of patients with AD. In AD, hyperphosphorylation of tau protein in neurons leads to microtubule depolarization, aberrant formation of tau tangles, and disruption of several cellular processes accompanied by impaired autophagy and damaged mitochondria.

There is growing evidence that autophagy flux is considerably impaired in AD animal models and patients with AD (Bordi et al., 2016; Fang et al., 2019b; Long et al., 2020; Figure 1). A notable ultrastructural abnormality in AD brains was initially shown to be an accumulation of autophagic vacuoles within dystrophic neurites (Hamano et al., 2021). Consequently, impaired autophagy has been observed in laboratory models of AD (Yang et al., 2011). Notably, mutations in autophagy-related genes recapitulated pathological neurodegenerative phenotypes in mice (Komatsu et al., 2006). Although autophagy protects cells from death by degrading toxic substances, excessive or imbalanced autophagy results in a condition termed “autophagic stress,” which contributes to cell death and neurodegeneration (Roney et al., 2021). Therefore, autophagy plays a unique role in AD pathogenesis.

Autophagy can be either non-selective or selective in the removal of damaged protein aggregates or specific organelles. Selective autophagy has also been identified as being involved in AD pathology (Guan et al., 2022). Among several subtypes of selective autophagy, mitophagy, which mediates the selective removal of mitochondria, has attracted increasing attention (Kerr et al., 2017). Healthy mitochondria are critical for the physiological function of neurons because of their ability to produce ATP, buffer calcium, and regulate ROS production; however, the accumulation of damaged mitochondria is detrimental to neurons (Chang and Reynolds, 2006; Devine and Kittler, 2018). Defective mitochondria can be cleared by mitophagy, which is initiated when the mitochondrial membrane potential is dissipated due to functional damage (Kerr et al., 2017). Mitophagy is essential for degrading dysfunctional mitochondria and maintaining mitochondrial homeostasis, which is critical for normal neuronal functions (Li et al., 2021). Mitochondrial dysfunction occurs with age (Chistiakov et al., 2014) and is exacerbated in age-related neurodegenerative diseases (Lane et al., 2015). Dysfunction of mitochondria with abnormal morphology reduced biogenesis, increased oxidative damage, reduced mitochondrial axonal transport, disturbed fission and fusion balance, and caused defective mitophagy. All of them are common features in brain tissues from AD animal models and postmortem patients with AD (Wang et al., 2020). Mounting evidence has shown that mitochondrial dysfunction occurs in the early stages of AD (Adlimoghaddam et al., 2019; Albensi, 2019). Therefore, elucidating mechanistic links between mitochondrial dysfunction and tauopathies will help in understanding disease pathogenesis and conducting in-depth explorations of translational implications.

In this study, to delineate the role of autophagy and mitophagy in tauopathy, we first searched PubMed using the terms autophagy/mitophagy/mitochondria and tau; subsequently, we described the involvement of autophagy or mitochondria in tauopathy, as well as the involvement of tau in regulating autophagy or mitochondrial dysfunction.

## Interactions between tau and autophagy

### Autophagy mediates tau degradation

Intracellular tau proteins are degraded by the ubiquitin-proteasome system and the autophagy-lysosomes pathway to maintain homeostasis (Schmidt et al., 2021). In a study exploring the role of the proteasome in tau degradation in rat primary neurons, the amount of tau was found to decrease rather than increase when proteasomal inhibitors were used (Boselli et al., 2017). The reduction in tau is possibly due to the compensatory upregulation of autophagy with increased levels of LC3-II and increased numbers of autophagosomes, which unexpectedly elucidated the importance of autophagy in tau clearance (Hamano et al., 2021). Studies have demonstrated that tau-enriched granules are detected in neuronal lysosomes of the human brain (Ikeda et al., 1998). Moreover, *in vitro* studies based on autophagy inhibitors have provided compelling evidence for the importance of autophagy in tau degradation (Silva et al., 2020). In a human neuroblastoma M1C cell line expressing inducible wild-type tau, treatment with the lyosomotrophic agent NH_4_Cl and the autophagy inhibitors chloroquine and 3-methyladenine (3MA) resulted in tau accumulation in the cells (Hamano et al., 2008). In inducible N2a cell lines overexpressing different tau constructs, inhibition of autophagy resulted in increased tau aggregation because both soluble and insoluble tau degradation are inhibited (Wang et al., 2009). Therefore, we inferred that the autophagic lysosomal system degrades soluble and insoluble tau.

**FIGURE 2:**
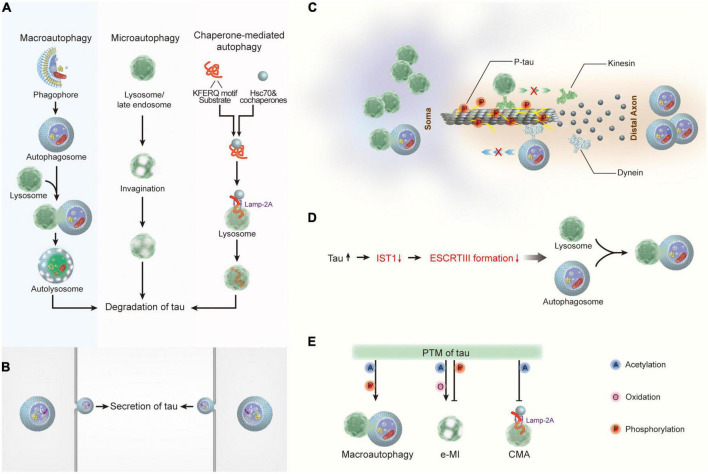
The crosstalk between tau and autophagy. **(A)** Macroautophagy, microautophagy, and chaperone-mediated autophagy are involved in tau degradation in neurons. **(B)** Tau protein can be secreted *via* the autophagy-based unconventional secretory pathway. **(C)** Pathological tau reduces its binding affinity to microtubules, thereby leading to microtubule depolymerization and the impairment of both the degradative lysosome transportation to axons and the retrograde transport of autophagosomes to lysosomes. **(D)** Tau accumulation disrupts autophagosome-lysosome fusion *via* inhibiting IST1 expression and disrupting ESCRT-III complex formation. **(E)** Post- translational modifications of tau regulate its degradation by autophagy. Hsc70, heat shock cognate 70; Lamp-2A, lysosome-associated membrane protein type 2A; ESCRT, the endosomal sorting complex required for transport; IST1, IST1 factor associated with ESCRT-III; e-MI, endocytic microautophagy; CMA, chaperone-mediated autophagy.

Studies have revealed that intracellular tau proteins can be degraded by at least three forms of autophagy: macroautophagy, CMA, and endocytic microautophagy (e-MI) (Hamano et al., 2021; Figure 2A). Considering that the 3MA and chloroquine used in the aforementioned studies are macroautophagy inhibitors, macroautophagy is the most studied type of autophagy involved in tau clearance (Caballero et al., 2021). Furthermore, studies targeting macroautophagy-related genes have provided direct evidence of this function. Aggresome-like tau inclusions are resistant to clearance when ATG5 or ATG7 is deleted (Wong et al., 2008; Inoue et al., 2012). In addition to tau aggregates, other forms of tau protein, including caspase 3 cleaved tau (D421), A152T mutant tau, acetylated tau at K174 and K274, and KXGS-phosphorylated tau, are degraded predominantly by macroautophagy (Wang et al., 2009; Dolan and Johnson, 2010). Studies have indicated that CMA and e-MI differentially contribute to the degradation of wild-type and pathogenic variants of tau (Xu et al., 2021). In contrast with macroautophagy, which is a non-selective bulk degradation process of cytoplasmic constituents, CMA is a selective autophagy process that selectively degrades substrate proteins *via* the KFERQ-like motif recognized by the cytosolic chaperone Hsc70 (Bourdenx et al., 2021). Tau also contains two KFERQ-like motifs, _336_QVEVK_340_ and _347_KDRVQ_351_, in its fourth repeat domain (Zeng et al., 2021). Unsurprisingly, CMA has been confirmed as one of the preferred pathways for the degradation of tau protein under physiological conditions (Caballero et al., 2018). Furthermore, CMA also contributes to the degradation of Tau_*RD*_ΔK280 (a truncated tau expressing the pro-aggregated repeat domain with the ΔK280 deletion mutation) and the A152T mutant tau (Wang et al., 2009; Caballero et al., 2018), both of which retain the KFERQ motifs. In addition to initiating CMA, Hsc70 also targets cytosolic proteins with this motif for degradation through e-MI. The role of e-MI in tau degradation has recently been examined. Although wild-type tau protein can be degraded through e-MI, it is much less than the amount degraded through CMA (Caballero et al., 2018). Surprisingly, pathological P301L-tau showed a significant impact on all three autophagic pathways, with almost undetectable levels of macroautophagy, CMA, and e-MI contributing to NFT formation in P301L-tau mice (Caballero et al., 2018). Thus, the results suggest that different tau species can be degraded by the three types of autophagy to different degrees.

### Autophagy mediates tau secretion

In addition to participating in the protein degradation process, autophagy mediates the secretion of cytosolic proteins (Cavalli and Cenci, 2020). Although further research is required to understand the secretory route regulated by autophagy, mounting evidence suggests that secretory autophagy facilitates the release of cytosolic proteins devoid of signal peptides (Gonzalez et al., 2020). Tau protein does not have an apparent signal peptide sequence; therefore, it cannot access the conventional protein secretory pathway through transportation from the endoplasmic reticulum to the Golgi body (ER-Golgi system) (Pernegre et al., 2019). Studies have demonstrated that human tau protein can be secreted into the extracellular space in various *in vitro* and *in vivo* systems. In various types of neuronal cells, including neuronal immortalized cell lines from humans (SH-SY5Y) (Karch et al., 2013) and mice (N2a) (Kanmert et al., 2015), mouse primary cortical neurons (Karch et al., 2012), and iPSC-derived human neurons (Kanmert et al., 2015), as well as non-neuronal cell lines such as HEK293T (Chai et al., 2012; Karch et al., 2012, 2013) and HeLa (Plouffe et al., 2012). Tau is present in full-length, truncated, or phosphorylated forms in the culture media derived from these cells, independent of cell death. In addition, tau protein was detected in the brain interstitial fluid (Barini et al., 2022) and cerebrospinal fluid (Cicognola et al., 2019) in several *in vivo* models of neurodegenerative diseases. In addition to type I unconventional secretion, the autophagy-mediated secretory pathway has been demonstrated as an important mechanism underlying tau secretion (Katsinelos et al., 2018; Figure 2B). Six tau isoforms arising from alternative splicing of MAPT (3R0N, 3R1N, 3R2N, 4R0N, 4R1N, and 4R2N) and phosphorylated tau (pT181, pS199, pT231, pS262, pS396, and pS356) are secreted from neuronal cells in an autophagy-dependent manner, indicating that the secretion of normal and pathological tau is mediated, at least to some extent, by autophagy (Kang et al., 2019). Although the function of extracellular tau remains unclear, studies have indicated that extracellular tau might be a critical factor in tauopathies because recombinant 4R2N tau in the conditioned medium of neuronal cells results in increased intracellular calcium concentrations and subsequent cell death (Sebastian-Serrano et al., 2018; Ruan et al., 2021). Therefore, in-depth exploration of autophagy-mediated tau secretion would improve the understanding of the mechanisms of aggregation and propagation of tau proteins.

### Tau acts as an autophagy regulator

Most research has mainly focused on the role of autophagy in the regulation of tauopathies in neurodegenerative disorders; thus, the impact of pathological tau on the function of autophagy remains unclear. According to the literature, the impact of tau on autophagy mainly has three aspects (Hamano et al., 2021): abnormal tau proteins inhibit the binding and assembly of microtubules and disrupt the cargo transport mediated by microtubules, which further impairs autophagy (Esteves et al., 2019). In contrast, tau accumulation disrupts autophagosome-lysosome fusion by disrupting ESCRT-III complex formation (Feng et al., 2020); furthermore, posttranslational modifications of tau participate in the regulation of tau degradation by autophagy (Guha et al., 2020).

Tau protein belongs to the family of microtubule-associated proteins, and up to 98% of tau binds to microtubules under physiological conditions (Igaev et al., 2014). Neurons are unique polarized cells with a highly extended long axon. In contrast with other MAPs, tau protein is predominantly localized in the axons of neurons (Wang and Mandelkow, 2016). Therefore, the dynamic processes of the binding and dissociation of tau from microtubules stabilize microtubule assembly and participate in the regulation of axonal transport. In neurons, mature lysosomes are largely dispersed in the soma (Tammineni et al., 2017), whereas autophagosomes are continually produced at the axon tip of neurons (Maday and Holzbaur, 2014). Carrying the cellular cargo destined for degradation, autophagosomes are retrogradely transported along microtubule tracks driven by dynein toward the soma, where they fuse with lysosomes to form autophagolysosomes (Maday and Holzbaur, 2012). Pathological changes in tau, such as tau hyperphosphorylation, reduce its binding affinity to microtubules, leading to microtubule depolymerization and impairment of axonal transport (Alonso et al., 2018; Figure 2C). Axonal transport disturbances can further impede autophagy in neurons, interrupting the autophagic clearance of inclusions (Weng and He, 2021). In addition, tau accumulation was found to cause autophagy deficits by suppressing IST1 expression, which interferes with the formation of the ESCRT-III complex, thus impeding lysosomal degradation (Feng et al., 2020; Figure 2D). As described, the reverse inhibition of autophagy by abnormal tau proteins appears to form a vicious cycle, resulting in enhanced neurodegeneration.

Finally, multiple types of post-translational modifications, including acetylation, oxidation, and phosphorylation, can enhance or weaken the clearance of tau through autophagy (Figure 2E). Acetylation of tau at K274 increases the interaction between tau and hsc70 (Haj-Yahya and Lashuel, 2018). Notably, acetylated tau is preferentially degraded by e-MI with high enrichment in LE/MVB and macroautophagy, and CMA activity is inhibited (Caballero et al., 2021). The effects of oxidation and phosphorylation on tau degradation by CMA and e-MI have also been examined (Wang et al., 2009). This study found a significant decrease in the association and internalization of C291A/C322A mutant tau in late endosomes by e-MI, suggesting that the oxidation of tau at C291/C322 is an important precondition for completing the internalization of tau through e-MI. In contrast, phosphorylation of tau in the microtubule-binding domain promotes its degradation by macroautophagy while reducing its degradation by e-MI (Caballero et al., 2018).

## Interactions between tau and mitochondria/mitophagy

### Tau contributes to mitochondrial dysfunction

*In vitro* and *in vivo* studies in cellular and animal models of tauopathy have revealed a spectrum of mitochondrial abnormalities caused by pathological tau proteins (Kerr et al., 2017). These abnormalities involve many aspects, including (1) mitochondrial bioenergetics, fulfilling high energy demands; (2) mitochondrial transport, removal of damaged mitochondria from axons; (3) mitochondrial fission and fusion, mediating mitochondrial morphology changes; and (4) mitophagy, leading to the selective removal of dysfunctional mitochondria. We have mainly concentrated on the last three elements in this section because the abnormality of mitochondrial bioenergetics has been explored (Patro et al., 2021).

**FIGURE 3:**
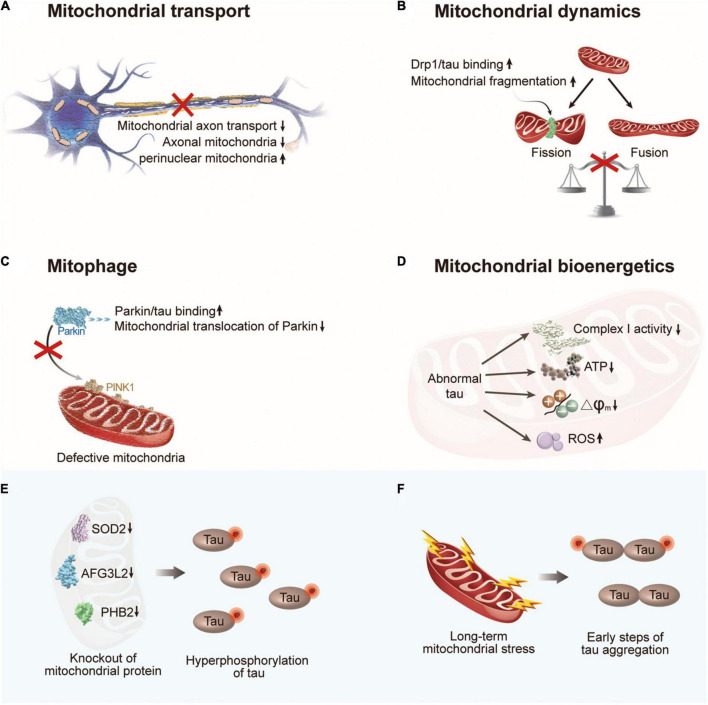
The crosstalk between abnormal tau and mitochondrial dysfunction. **(A)** Abnormal tau impairs axonal transport of mitochondria with a decrease in the number of axonal mitochondria and an increase in perinuclear mitochondria. **(B)** Abnormal tau disturbs mitochondrial fission/fusion dynamics through interacting with Drp1, resulting in enhanced mitochondrial fission. **(C)** Abnormal tau impairs mitophagy by interacting with Parkin to prevent Parkin translocation to defective mitochondria. **(D)** Abnormal tau impairs mitochondrial bioenergetics *via* inhibiting complex I activity, reducing ATP levels and ΔΨm, and increasing ROS generation. **(E)** Hyperphosphorylation of tau can be induced by deficiency of mitochondrial protein SOD2, AFG3L2, or PHB2. **(F)** Long-term mitochondrial stress induces tau dimerization. Drp1, dynamin-related protein 1; PINK1, PTEN-induced putative kinase 1; SOD2, superoxide dismutase 2; AFG3L2, AFG3 like matrix AAA peptidase subunit 2; PHB2, prohibitin 2.

Efficient delivery of mitochondria is critical for neurons to fulfill their high energy requirements. As motor proteins, such as kinesin and dynein, travel along the microtubules, mitochondria are delivered to neurons (Sheng, 2014). The transport and distribution of mitochondria are governed by microtubule networks maintained by tau (Guo et al., 2017; Figure 3A). The overexpression of wild-type tau increased the pausing frequency of mitochondrial movement by 17% in neurons and decreased the velocity of mitochondria in the axons. Additionally, hyperphosphorylated tau protein at sites Ser199/Ser202/Thr205 increased the pausing frequency of mitochondrial movement by up to 33% and suppressed mitochondrial movement in axons to a greater degree than wide tau by increasing the inter-microtubule distance (Shahpasand et al., 2012). Moreover, studies have indicated a significant increase in the angle that defines the orientation of mitochondria in the axons of neurons expressing P301L tau, which might increase mitochondrial fusion and change the number of mitochondria in the axons (Schulz et al., 2012; Rodriguez-Martin et al., 2016). Decreased numbers of axonal mitochondria and increased numbers of perinuclear mitochondria have been observed in neuronal models that express pathological forms of tau (Perez et al., 2018; Szabo et al., 2020). These investigations show that different degrees of impairment in mitochondrial axonal transport can be caused by wild-type, P301L mutant, and hyperphosphorylated tau.

Tauopathies not only impair the axonal transport of mitochondria but also disturb mitochondrial fission/fusion dynamics (Reddy and Oliver, 2019). Mitochondrial fission, followed by selective fusion, provides a mechanism for the removal of damaged and dysfunctional mitochondria. In mammalian cells, mitofusin1 (Mfn1), Mfn2, and optic atrophy1 (Opa1) are required for mitochondrial fusion, whereas dynamin-related protein 1 (Drp1) and mitochondrial fission protein 1 (Fis1) are involved in this process (Chan, 2020). According to the literature, mitochondrial fusion maintains mitochondrial function by enabling the exchange of contents, whereas excessive mitochondrial fission worsens mitochondrial function and is regarded as an indication of dysfunction (Chen and Chan, 2010; Adebayo et al., 2021). Wild-type tau has been shown to increase the expression of fusion proteins Mfn1, Mfn2, and Opa1 and prevent the localization of Drp1 to mitochondria without altering its expression level (DuBoff et al., 2012; Li et al., 2016; Perez et al., 2018). In contrast, the interaction between hyperphosphorylated tau and the fission protein Drp1 promotes mitochondrial fission and excessive mitochondrial fragmentation (Manczak and Reddy, 2012; Figure 3B).

Similar to the dynamic process of mitochondrial fission and fusion, mitophagy is a major mechanisms of mitochondrial quality control (Pickles et al., 2018). Regarding the important role of the PINK1/Parkin pathway in basal mitophagy (Whitworth and Pallanck, 2017), studies have explored the effect of tau on PINK1/Parkin mitophagy. Notably, human NH_2_-tau fragment mapping between 26 and 230 amino acids of the full-length protein was found to be associated with selective mitochondrial autophagic clearance in primary neurons, which increased the autophagic turnover of mitochondria (Amadoro et al., 2014). However, this effect was not observed for full-length wild-type tau. In contrast, intracellular accumulation of wild-type tau resulted in mitophagy deficits *in vitro* and *in vivo* (Hu et al., 2016). The underlying mechanisms of the effect of wild-type tau and FTD (P301L) mutant tau on mitophagy have been discussed in neuroblastoma cells and the *Caenorhabditis elegans* nervous system (Cummins et al., 2019). By interacting with Parkin and trapping it in the cytosol, both tau species have been shown to impede the translocation of Parkin to defective mitochondria (Cummins et al., 2019; Figure 3C). In addition to PINK1/Parkin-mediated mitophagy, other receptors, such as BNIP3L/NIX, have been implicated in mitophagy control (Villa et al., 2018). One study has shown that BNIP3L/NIX is downregulated in some patients; however, whether tau is responsible for the suppression of BNIP3L/NIX remains unclear (Fang et al., 2019b). Last but not least, abnormal tau also impairs mitochondrial bioenergetics via inhibiting complex I activity, reducing ATP levels and ΔΨm, and increasing ROS generation (Figure 3D).

### Dysfunctional mitochondria induce tauopathies

From another perspective, the abnormal accumulation of dysfunctional mitochondria in neurons has been shown to induce tauopathies through various mechanisms (Figures 3E,F). Hyperphosphorylation of tau was induced by the chronic generalized inhibition of mitochondrial complex I in rotenone-treated rats (Hoglinger et al., 2005). Another study linked mitochondrial dysfunction to tau hyperphosphorylation in mitochondrial SOD2-deficient mice (Melov et al., 2007). Similarly, AFG3L2 deficiency, a component of the mitochondrial m-AAA complex, promotes the fragmentation of the mitochondrial network, impairs mitochondrial transport, and increases the phosphorylation of tau in neurons (Kondadi et al., 2014). Subsequent studies have demonstrated that mitochondrial dysfunction can also trigger tau aggregation. In mitochondrial prohibitin-2-deficient mice, hyperphosphorylation and NFT formation of tau have been observed in the hippocampus, although the precise mechanism is unknown (Merkwirth et al., 2012). A recent study indicated that the early steps of tau aggregation can be induced by long-term mitochondrial stress by affecting oxidative balance and cellular proteostasis (Samluk et al., 2022). Notably, an increase in tau phosphorylation was not a prerequisite for dimerization under these conditions. These observations are consistent with the discovery that modulating mitochondrial protein import activity stimulated the clearance of protein aggregates in the cytosol (Nowicka et al., 2021; Schlagowski et al., 2021), indicating a potential connection between mitochondrial dysfunction and tau aggregation.

Another important association between mitochondria and tauopathies is oxidative stress. Mitochondria are considered “professional” producers of ROS, where ROS are produced permanently as a by-product of mitochondrial electron transport chains (Alavi Naini and Soussi-Yanicostas, 2015). Increased ROS levels are associated with aging and have been identified as a trigger of AD pathogenesis (Stefanatos and Sanz, 2018). Evidence has suggested that ROS accumulation plays a critical role in tau hyperphosphorylation. The activity of tau kinases, such as GSK3β, CDK5, p38 mitogen-activated protein kinase (MAPK) (Hugon and Paquet, 2021) and CaMKII (Oka et al., 2017), are upregulated under oxidative stress (Zhu et al., 2000; Alavi Naini and Soussi-Yanicostas, 2015; Anderson, 2015). In contrast, ROS inhibited PP2A, the major tau phosphatase (Liu et al., 2015). Thus, tau hyperphosphorylation is possibly induced by oxidative stress through these kinases and phosphatases. Another possible link between ROS and pathologic tau phosphorylation is asparagine endopeptidase (AEP). As a lysosomal cysteine protease (Zhang et al., 2017b), AEP directly cleaves tau, and the resultant fragments facilitate tau hyperphosphorylation and aggregation (Zhang et al., 2014; Wang et al., 2018). Moreover, the knockout of AEP prevents tau hyperphosphorylation in P301S mice (Zhang et al., 2016; Zhang et al., 2020). AEP is up-regulated during aging (Chen et al., 2021) and can be activated by oxidative stress (Ahn et al., 2021) further highlights the important role of oxidative stress in tau pathology.

In addition to the aforementioned points, we emphasize the important role of Ca^2+^ signaling in mitochondrial dysfunction and tau hyperphosphorylation. Mitochondrial function and Ca^2+^ homeostasis are intertwined processes (Bravo-Sagua et al., 2017). Under physiological conditions, Ca^2+^ plays an active role in regulating mitochondrial function, and calcium homeostasis disturbance can disrupt normal mitochondrial function and increase oxidative stress (Tong et al., 2018). Phosphorylation of the tau protein is also greatly affected by cytoplasmic Ca^2+^ because the activity of major kinases responsible for tau phosphorylation is dependent on Ca^2+^. Calcium homeostasis disturbance has been demonstrated to activate GSK3β and other tau kinases, including CDK5 and CaMKII, *via* the Ca^2+^-dependent protease calpain, resulting in abnormal hyperphosphorylation of tau (Feng et al., 2013; Mahaman et al., 2019; Liu et al., 2021).

## Neuroinflammation in tauopathy

Neuroinflammation is the third core neuropathological feature of the AD brain in addition to Aβ plaques and NFTs (Calsolaro and Edison, 2016). Persistent neuroinflammation has been observed in the early stages of AD (Bradburn et al., 2019). Such neuroinflammatory alterations have been observed in the postmortem brains of patients with AD, as well as in animal models (Newcombe et al., 2018). Activated astrocytes and microglia are characteristically found in areas of pathological protein deposition (Sardi et al., 2011). Elevated levels of a number of pro-inflammatory cytokines or inflammatory markers have also been discovered (Kamer et al., 2008).

### Neuroinflammation and autophagy

Inflammation, autophagy, and AD are linked processes. Autophagy and inflammation are two biological processes upregulated by cells in response to a wide range of stressful events (Wu and Adamopoulos, 2017). Notably, these two pathways interact and regulate each other bidirectionally (Deretic, 2021). Different cytokines regulate the autophagy of certain pro-inflammatory cytokines; for example, IFN-γ, TNF-α, IL-1, IL-4, IL-17, and IL-6 can activate cellular autophagy, and this process can be blocked by IL-10, IL-33, and IL-37 (Harris, 2011; Ge et al., 2018). It has been reported that IFN-γ and TNF-α induced autophagy by blocking the p38 MAPK-NF-κB pathway (Ye et al., 2011; Matsuzawa et al., 2012); IL-1α and IL-1β significantly increased LC3 mRNA expression, LC3-I and LC3-II protein levels, and autophagic flux (Okutsu et al., 2021); IL4-induced autophagy depends on JAK signaling *via* an mTOR-independent, PtdIns3K-dependent pathway (Xia et al., 2018); IL-6 activated autophagy through the IL-6/JAK2/BECN1 pathway (Hu et al., 2021); and IL-17A mediated excessive neuronal autophagy through the Src-PP2B-mTOR pathway (Liu et al., 2019); IL-10 inhibited autophagic flux through the PI3K/Akt signaling pathway (Wang et al., 2014); IL-33 prevented traumatic brain injury-induced elevation of IL-1β and TNF-α levels and upregulation of autophagy (Gao et al., 2018); and transfection of human monocyte line THP-1 with IL-37β decreased mTOR expression (Nold et al., 2010). In contrast, autophagy plays a key role in inflammation by affecting the development, homeostasis, and survival of inflammatory cells and influencing the transcription, processing, and secretion of many cytokines, such as IL-1β, IL-6, and TNF-α (Wu et al., 2016). Autophagy induction in microglia can alleviate neuroinflammation by degrading misfolded proteins, damaged mitochondria, and activated inflammasomes (Su et al., 2016). Thus, impaired autophagy is associated with persistent tissue inflammation and is possibly a mechanism in the pathogenesis of chronic inflammation (Qian et al., 2017).

### Neuroinflammation and mitophagy

Healthy mitochondria partially regulate the immune response and reduce inflammatory signaling, but damaged mitochondria lead to the activation of the NF-κB pathway and NLRP3 inflammasome, increasing the release of ROS and cardiolipin (Elliott and Sutterwala, 2015; Fang et al., 2019b). The NF-κB pathway is further activated by increased ROS levels, leading to the upregulation of pro-inflammatory cytokines, such as TNF-α and IL-1β, and exacerbates the inflammatory response (Blaser et al., 2016). In contrast, mitochondrial autophagy ameliorates inflammation by removing damaged mitochondria and reducing the downstream cascade response induced by damaged mitochondria (Green et al., 2011). The accumulation of damaged mitochondria and mitophagy dysfunction in AD affects neurons and microglia. Researchers have found that the accumulation of damaged mitochondria and defective mitophagy in microglia results in the impaired phagocytic clearance of Aβ plaques in an APP/PS1 mouse model (Fang et al., 2019b). Enhanced mitophagy increased the efficiency of microglia phagocytosis while attenuating NLRP3/caspase-1-dependent neuroinflammatory responses (Ahmed et al., 2021).

### Tau and neuroinflammation

Tauopathies are also closely associated with neuroinflammation and microglia/astrocyte activation (Leng and Edison, 2021). Age-dependent astrocyte proliferation/microglial activation and pathological neuroinflammatory changes have been observed in the CNS of different tauopathy models in the early stage of disease without neuronal loss, showing activated CD68-positive microglia around neurons containing hyperphosphorylated tau NFTs (Maphis et al., 2015; Laurent et al., 2017; Van Zeller et al., 2021). *In vitro* studies have shown that oligomers and fibers of tau induce morphological changes in microglia and upregulate the expression of pro-inflammatory cytokines (IL-6, IL-1β, TNF-α) *via* the NF-κB and MAPK signaling pathways (Kovac et al., 2011; Sun et al., 2021).

However, neuroinflammatory response is thought to influence tau pathogenesis. Microglia were also observed to be involved in the diffusion of tau proteins (Spanic et al., 2019). Aggregates of tau can be released into exosomes and spread between cells or tissues in a prion-like propagation manner when microglia in the CNS cannot adequately convert tau into a non-toxic form (Stancu et al., 2019; Amro et al., 2021). Moreover, extracellular aggregates of tau can activate the NLRP3 inflammasome (Stancu et al., 2019), leading to microglial activation and the elevation of pro-inflammatory cytokines, further impairing the ability to process tau aggregates (Ising et al., 2019). Thus, the prion-like propagation of tau aggregates and the activation of the NLRP3 inflammasome can trigger a vicious cycle of sustained microglial hyperactivation and severe inflammatory responses, which is essential for the development and progression of AD (Bai and Zhang, 2021).

## Therapeutic strategies targeting autophagy, mitophagy and neuroinflammation

Although AD is the most prevalent neurodegenerative disorder worldwide, no effective treatment is available. Over 100 medications are currently undergoing clinical trials, with the majority focusing on Aβ and tau metabolism, inflammation, neurotransmitter receptors, and synaptic plasticity. In this study, we focused on AD therapeutic strategies that target autophagy, mitophagy, and neuroinflammation.

### Therapeutic strategies targeting autophagy

Studies have suggested that autophagy activation can induce enhanced degradation of aggregated proteins and damaged organelles, which might be ideal for AD therapy (Li et al., 2017). Because autophagy is a highly dynamic and intricate cellular process, it could theoretically be induced *via* multiple pathways with a range of pharmacological targets for the development of corresponding agonists or antagonists (Uddin et al., 2018; Table 1).

**TABLE 1:** Alzheimer’s disease **(AD)** therapeutic strategies targeting autophagy.

Target	Classification	Drug	Mechanism	Therapeutic effects	Status	References
Autophagy	mTOR inhibition	Rapamycin	Selective inhibitors of TORC1	Rescues the mitochondria abnormality and cognitive impairment	Preclinical research	Ding et al., 2021
		Curcumin	Inhibits the PI3K-Akt-mTOR signaling pathway	Improves the spatial learning and memory capability	Preclinical research	Ordonez-Gutierrez and Wandosell, 2020
		Carbamazepine	Enhances the autophagic flux	Improves the spatial memory of 3 × Tg AD mice	Preclinical research	Zhang et al., 2017a
	AMPK activation	Resveratrol	AMPK and sirtuin 1 activation	Attenuates brain amyloidosis in APP/PS1 mice	Preclinical research	Vingtdeux et al., 2010
		Lithium	Inositol monophosphate dependent activation	Attenuates tauopathies in 3 × Tg mice	Preclinical research	Matsunaga et al., 2015
	mTOR and AMPK independent manner	Corynoxine B	Up-regulates LC3-II	Promotes the clearance of mutant tau aggregation in P301L-tau mice	Preclinical research	Durairajan et al., 2022
		Curcumin analog C1	Induces lysosomal biogenesis	Improves synaptic and cognitive functions in 5×FAD, Tau P301S and 3 × Tg mice	Preclinical research	Song et al., 2020
	Others	Tat-beclin 1	Increase autophagic flux *via* the canonical pathway	Decreases the accumulation of polyglutamine expansion protein aggregates	Preclinical research	Shoji-Kawata et al., 2013
		Overexpression of TFEB	Enhance autophagy *in vivo*, reduce levels of PHF-tau	Reverse the deposition of lipofuscin granules and memory deficits	Preclinical research	Wang et al., 2016

Most chemical inducers of autophagy act by inhibiting mTOR or by activating AMPK. Additionally, an FDA-approved drug for AD has been shown to enhance mTOR-dependent or independent autophagy (Hirano et al., 2019). Rapamycin and its derivatives are selective inhibitors of the target of rapamycin complex 1 and modulators of the mTOR pathway (Kim and Guan, 2015). Experimental results from animal models suggest that rapamycin alleviates Aβ aggregation and tauopathies and improves memory dysfunction (Li et al., 2017). In clinical trials, low doses of rapamycin improved some indicators of aging (Selvarani et al., 2021). Curcumin, a natural polyphenolic compound, can effectively inhibit the PI3K-Akt-mTOR signaling pathway (Khan et al., 2020). In the APP/PS1 transgenic AD mouse model, curcumin improved spatial learning and memory capability by inducing autophagic degradation of Aβ aggregates (Chainoglou and Hadjipavlou-Litina, 2020; Ordonez-Gutierrez and Wandosell, 2020). Resveratrol, another plant-derived polyphenol, exerts beneficial effects against AD-like pathology through AMPK-mediated activation of autophagy (Yan et al., 2020). Additionally, resveratrol can activate sirtuin 1, leading to enhanced autophagy, increased oxidative/reductive NAD, accelerated removal of aberrant proteins, and increased neuronal survival (Deng et al., 2019). Notably, several antipsychotic drugs have been shown to exert autophagy-inducing effects. The mood stabilizer lithium has been reported to promote autophagy by activating AMPK and significantly ameliorates tauopathies in 3 × Tg AD model mice (Matsunaga et al., 2015). Similarly, the antiepileptic medication carbamazepine has autophagy-inducing roles, which significantly enhanced autophagic flux in APP/PS1 mice (Li et al., 2013). Importantly, this drug protected against memory impairment and increased the Aβ content in a mouse model of AD (Zhang et al., 2017a).

Plant-derived autophagy enhancers that induce autophagy in an mTOR- and AMPK- independent manner have recently attracted attention. Corynoxine B and curcumin analog C1 are two promising plant-derived natural compounds for the treatment of AD that act as inducers of autophagy (Durairajan et al., 2022). The underlying pharmacological mechanisms were examined, and corynoxine B was found to induce autophagy by upregulating LC3-II (Lu et al., 2012). Similarly, curcumin analog C1 reduced tau aggregates by inducing lysosomal biogenesis in 5×FAD, Tau P301S, and 3 × Tg mice (Song et al., 2020).

In addition to small-molecule therapies, other strategies can be used to enhance autophagy. One study linked the HIV-1 Tat protein transduction domain to a modified 18 amino acid sequence based on residues 267-284 of Beclin 1. The hybrid peptide was found to increase autophagic flux *via* the canonical pathway (Shoji-Kawata et al., 2013). Gene therapy is a promising therapeutic strategy. Overexpression of transcription factor EB (TFEB), the main regulator of the autophagy-lysosome pathway, could enhance autophagy *in vivo*, reduce levels of tau, and thereby reverse memory deficits in P301S mice (Wang et al., 2016). Studies have also explored the application of fluorescent probe techniques for the detection, monitoring, and treatment of AD by modulating autophagy (Iyaswamy et al., 2022; Wang et al., 2022).

### Therapeutic strategies targeting mitophagy

Because mitochondrial damage is a characteristic of several neurological disorders, including AD, enhancing the removal of defective mitochondria and their contents may exert potential therapeutic benefits (Kerr et al., 2017; Table 2). Among the many potent mitophagy inducers identified in cellular and animal models, NAD^+^ precursors, urolithin A, actinonin and spermidine have been demonstrated to be effective in extending health span and protecting neurons in animal models and human cells (Lou et al., 2020).

**TABLE 2:** Alzheimer’s disease (AD) therapeutic strategies targeting mitophagy.

Target	Classification	Drug	Mechanism	Therapeutic effects	Status	References
Mitophagy	NAD^+^ precursors	Nicotinamide ribose	Repairs the disabled mitochondria	Improves the short-term spatial memory of aged mice, and the contextual fear memory of AD mice	Preclinical research	Xie et al., 2019
		Nicotinamide adenine	Repairs the disabled mitochondria	Improves cellular homeostasis based on association with dietary requirements	Preclinical research	Braidy et al., 2018
		Nicotinamide mononucleotide	Induces mitophagy	Abolishes AD-related tau hyperphosphorylation and reverses memory impairment in transgenic tau nematodes and mice	Preclinical research	Fang et al., 2019b
	Mitophagy enhancer	Urolithin A	Induces expression of mitophagy proteins and attenuates neuroinflammation	Alleviates APP and BACE1 expressions, Tau phosphorylation, Aβ deposition, and cognitive impairment	Preclinical research	Jayatunga et al., 2021; Lee et al., 2021
		Actinonin	Promotes the efficient removal of dead and/or damaged mitochondria	Reverses cell survival and defective mitochondrial respiration	Preclinical research	Kshirsagar et al., 2021
		Spermidine	Induces autophagosome formation	Positive impact on memory performance in older adults with subject cognitive decline	Phase II clinical trail	Wirth et al., 2018
		Nilotinib	Activation of Parkin	Increases soluble parkin leading to amyloid clearance and cognitive improvement	Phase II clinical trail	Lonskaya et al., 2013
	Gene therapy	Overexpression of PINK1	Activation of ALP	Promotes degradation of abnormal accumulated tau	Preclinical research	Jiang et al., 2021
		Silencing of miR-204	STAT3 pathway activation	Suppresses ROS production and mitochondrial autophagy in AD	Preclinical research	Zhang et al., 2021b

The major NAD^+^ precursors include nicotinamide, nicotinamide mononucleotides, and nicotinamide riboside. The effects of nicotinamide may be mediated by the modulation of sirtuin 1 activity and proton gradients that promote the acidification of lysosomes or autophagic lysosomes, resulting in the reduced accumulation of autophagic vesicles (Fang et al., 2019a). Supplementation with NAD^+^ precursors in animal models of AD can inhibit Aβ and tauopathies, increase the activity of PI3K-AKT and MAPK/ERK1/2 *via* NAD^+^-dependent SIRT1 and SIRT3, and eventually reverse cognitive impairment in 3 × Tg and APP/PS1 models (Hosseini et al., 2021; Wang et al., 2021).

Urolithin A and actinonin have been shown to trigger mitophagy in a PINK1/Parkin/NIX-dependent manner in AD animal models. These drugs ameliorate several pathological features of AD, including memory and learning deficits and Aβ/tau-associated aggregation (Kshirsagar et al., 2021, 2022). In addition, neuroinflammation can be inhibited by urolithin A by reducing the production of pro-inflammatory cytokines, such as TNF-α and IL-6, and increasing the level of anti-inflammatory IL-10 (Fang et al., 2019b; Gong et al., 2019).

Spermidine alleviates memory impairment and promotes longevity in *Drosophila*, *C. elegans*, and mouse models (Schroeder et al., 2021). Studies have suggested that spermidine induces mitochondrial autophagy through mTOR inhibition, AMPK activation (Yang et al., 2020), and ATM-dependent PINK1/Parkin signaling (Qi et al., 2016).

Mitophagy enhancement through Parkin activation is another promising strategy. Nilotinib, a tyrosine kinase inhibitor, may enhance Parkin recycling through the proteasome system (Lonskaya et al., 2014). Chronic treatment with nilotinib in APP transgenic mice enhanced Aβ clearance by increasing the interaction between Parkin and Beclin 1 (Lonskaya et al., 2013). Moreover, PINK1 overexpression effectively promotes the degradation of tau, rescuing neuronal loss, synaptic damage, and cognitive impairments in a mouse model of tauopathy (Jiang et al., 2021). Various non-transcribed microRNAs are involved in mitophagy, and Zhang et al. (2021b) demonstrated that downregulation of miR-204 reduced Aβ 1–42-induced mitochondrial damage, along with ROS production and mitochondrial autophagy *in vivo* and *in vitro*.

### Therapeutic strategies targeting neuroinflammation

The increased levels of inflammatory markers observed in patients suggest the involvement of neuroinflammation in the pathology of AD. From a therapeutic perspective, the use of non-steroidal anti-inflammatory drugs is significantly associated with a reduced risk of AD (Heneka et al., 2015). Therefore, anti-neuroinflammatory therapy has been widely studied for AD. Treatment strategies targeting neuroinflammation mainly comprise two aspects: microglia modulators and astrocyte modulators (Dhapola et al., 2021; Table 3).

**TABLE 3:** Alzheimer’s disease (AD) therapeutic strategies targeting neuroinflammation.

Target	Classification	Drug	Mechanism	Therapeutic effects	Status	References
Neuroinflammation	Microglia modulator	Thymoquinone	TLR4 inhibitor	Removes Aβ plaques and restores neuron viability	Preclinical research	Elibol et al., 2020
		TAK-242	Specific TLR4 signaling inhibition	Promotes a microglial switch from the inflammatory M1 phenotype to the protective M2 phenotype	Preclinical research	Cui et al., 2020
		GW2580	CSF1R inhibitor	Improves performance in memory and behavioral tasks and a prevention of synaptic degeneration	Preclinical research	Olmos-Alonso et al., 2016
		PLX3397	CSF1R inhibitor	Dramatic reduction of both intraneuronal amyloid as well as neuritic plaque deposition	Preclinical research	Sosna et al., 2018
	Astrocyte modulator	Stattic	STAT3 inhibitor	Rescues the activation of astrocytes in 5×FAD mice as well as impairments in learning and memory	Preclinical research	Choi et al., 2020
		MW181	p38 MAPK inhibitor	Reduces tau phosphorylation and Sarkosyl-insoluble tau aggregates	Preclinical research	Maphis et al., 2016
		MRS2179	P2Y1R inhibitor	Protects from the decline of spatial learning and memory	Preclinical research	Reichenbach et al., 2018

Microglial activation is considered a hallmark of neuroinflammation and is associated with the lipoprotein E signaling pathway (Serrano-Pozo et al., 2021). Triggering receptors expressed on myeloid cells (TREM2), Toll-like receptors (TLRs) and colon-stimulating factor-1 receptors (CSF1R) are considered important regulators of microglia (Hoogland et al., 2015; Nguyen et al., 2020). Studies have indicated that increased TREM2 expression improves memory performance in 5×FAD mice, whereas TREM2 deficiency exacerbates Aβ pathology in advanced disease stages (Yeh et al., 2017; Delizannis et al., 2021). In addition, several TLR4 inhibitors, including thymoquinone and TAK-242, ameliorated cognitive deficits and enhanced phagocytosis of BV2 microglia in animal models of AD (Cascella et al., 2018; Cui et al., 2020). Moreover, the selective CSF1R inhibitor GW2580 improved short-term memory and behavioral impairments by preventing microglial proliferation in APP/PS1 mice (Soto-Diaz et al., 2021). Long-term administration of the CSF1R inhibitor PLX3397 reversed spatial and emotional memory deficits in 5×FAD mice (Sosna et al., 2018).

Astrocyte reaction impairs the clearance of Aβ and tau proteins in the CNS. The Janus kinase/signal transducer and activator of transcription 3 (JAK/STAT3), calcineurin/nuclear factor of activated T cells (calcineurin/NFAT), nuclear factor-κB/nod-like receptor family pyrin domain containing 3 (NF-κB/NLRP3), MAPK, and P2Y1 purinoreceptor (P2Y1R) pathways are all involved in astroglial activation (Colombo and Farina, 2016; Giovannoni and Quintana, 2020). Intraperitoneal injection of Stattic, a selective STAT3 inhibitor, rescued astrocyte activation and impaired learning and memory in 5×FAD mice (Choi et al., 2020). MW181, a small-molecule inhibitor of p38α MAPK with isoform-selective, brain-permeable, and orally bioavailable properties, reduced the expression of IFN-γ and IL-1β, as well as tau phosphorylation and aggregation (Maphis et al., 2016). Furthermore, the P2Y1R antagonist MRS2179 has been shown to protect against the deterioration of spatial learning and memory in APP/PS1 mice (Reichenbach et al., 2018).

## Conclusion

The prevalence of AD, the most common neurodegenerative disease worldwide, is significantly increasing owing to the aging population (GBD 2019 Dementia Forecasting Collaborators, 2022). Lifestyles such as caloric restriction, intermittent fasting, and exercise have been reported to be beneficial for patients with AD (De la Rosa et al., 2020). However, no method is available to prevent, stop, or reverse AD. Aβ and tau interact closely and contribute to the pathology of AD. Aβ accelerates tau hyperphosphorylation by inducing the activation of CDK-5 and GSK-3β (Terwel et al., 2008; Hernandez et al., 2009). In addition to the promotion of tau phosphorylation, Aβ interferes with tau oligomerization and aggregation (Gamblin et al., 2003). Moreover, Aβ and tau play an important role in the activation of microglia and astrocytes in the brains of patients with AD (Zhang et al., 2021a). Aβ and tau are also jointly involved in autophagy and mitophagy dysfunction in AD pathology (Fang et al., 2019b; John and Reddy, 2021). Many studies on AD treatment have focused on pathological markers, Aβ, and tau, and immunotherapy has become the most used approach in these two areas (Weiner and Frenkel, 2006; Lemere, 2013). However, because immunotherapeutic agents targeting the clearance of Aβ and tau proteins have failed in the phase III clinical stage, the target of intervention has gradually shifted from specific pathological markers to complex mechanisms such as autophagy, mitophagy, neuroinflammation, and neurodegenerative processes of disease development (Levey, 2021; Srivastava et al., 2021; Stoiljkovic et al., 2021). Substantial progress has been observed in the study of autophagy and mitophagy in patients with AD. Therefore, deciphering the crosstalk among autophagy, mitophagy, neuroinflammation, and tauopathies is important to provide new insights into the molecular mechanisms and potential therapeutic strategies for AD.

This review systematically summarizes the knowledge of relationships among autophagy, mitophagy, and tauopathies, as well as the crosstalk between these processes and neuroinflammation. Abnormal tau affects nearly all aspects of mitochondrial functions, including the interrupted axonal transport of mitochondria, the disturbance of mitochondrial fission/fusion dynamics, and impaired mitophagy and mitochondrial bioenergetics. Additionally, tau protein acts as a versatile autophagy regulator, controlling processes from lysosome and autophagosome transportation and autophagosome-lysosome fusion to autophagy-mediated degradation. Autophagy is also involved in tau degradation and secretion. Dysfunctional mitochondria can also induce tauopathies. Interventions that activate autophagy or mitophagy can preserve synaptic plasticity and cognitive function. Importantly, autophagy, mitophagy, and tauopathies regulate neuroinflammation bidirectionally. Our analyses suggest that autophagy and mitophagy act both upstream and downstream of tauopathies and neuroinflammation, which constitutes a vicious cycle that enhances neurodegeneration in AD. Notably, most studies on autophagy and mitophagy pathway in the CNS have been performed on neurons. However, the autophagy and mitophagy pathways in other cell types, such as astrocytes and microglia, as well as the crosstalk between autophagy and mitophagy among different cell types, are largely unknown. Exploring these issues in detail would help to understand the pathophysiology of AD and develop therapeutic interventions. From the perspective of clinical therapeutics, we summarized the strategies and advances in drug development targeting autophagy, mitophagy, and neuroinflammation. Although the induction of autophagy, mitophagy, and anti-neuroinflammatory therapy has shown beneficial effects in AD, further clinical studies based on larger samples of human patients are necessary because the experimental evidence has been obtained mainly from preclinical studies.

## Author contributions

XL, MY, and LM conceived the original draft. XL wrote the original manuscript and prepared the figures. MY and LM edited the final manuscript and prepared the tables. All authors approved the submitted version.
